# Thyroid Embryonic Anomalies Involving the Medial and Lateral Anlagen: Two Surgical Case Reports

**DOI:** 10.1155/2019/3174848

**Published:** 2019-12-05

**Authors:** Ferdinand Rico, John Lung

**Affiliations:** Department of Surgery, Texas Tech University Health Sciences Center, 1400 S Coulter, Amarillo, TX, USA 79106

## Abstract

**Introduction:**

An ectopic anomalous accessory thyroid is extremely rare. We present two related case reports.

**Case:**

A 43-year-old morbidly obese female presented with a palpable left thyroid mass. Initial impression after preoperative workup was a diffuse bilateral multinodular goiter with a left nodule suspicious for malignancy. She underwent a total thyroidectomy. On further palpation after initial excision, two masses on the left neck area were noted, dissected, and excised. In the second case, a 65-year-old female presented with a substernal exophytic mass displacing the trachea to the right after a left thyroid lobectomy 4 years ago. Cytology revealed a retained substernal thyroid. She is scheduled for a substernal thyroidectomy through a median sternotomy.

**Discussion:**

In all partial or complete thyroidectomy cases, care should be taken to palpate or to use intraoperative ultrasound (IOUS) for any extra thyroid mass. An intuitive surgical approach is needed to evaluate for possible embryologic anomalies of the thyroid. There are two thyroid anlagen that can explain ectopic aberrant anomalies: the median anlage-thyroid rest and the lateral anlage from the ultimobranchial body of the fourth pharyngeal pouch. Surgeons should keep in mind that multiple ectopic thyroid glands could be present in any thyroidectomy procedure.

## 1. Introduction

The thyroid gland develops from the median bud of the pharynx from the foramen cecum to the thyroid isthmus. An ectopic thyroid tissue is where the thyroid gland is located other than anterior to the trachea in the lower neck due to aberrations in embryogenesis [1]. Most patients with ectopic thyroids do not have symptoms, but obstructive symptoms and hypothyroidism have been reported in cases [2]. The prevalence of an ectopic thyroid is estimated around 1 per 100,000 to 300,000 people and occurs in 1 in 4,000-8,000 people with thyroid disease [3]. Ectopic thyroid tissue lateral to the normal midline location is rare. The exact definition of lateral ectopic thyroid tissue is not defined consistently in the literature with some reports defining it as lateral to the carotid sheath and jugular vein [4]. Others define it as an ectopic thyroid tissue embedded in or deep to the strap muscles that appears off the midline [5]. We present a case of a morbidly obese patient with an enlarged heterogeneous left lobe of the thyroid on imaging that had two ectopic masses near the left thyroid and a right persistent thyroid rest. The second case depicts a possible retained or a missed substernal thyroid that was accidentally discovered after a prior left thyroid lobectomy 4 years ago. We discuss the preoperative workup, surgical anatomy, postoperative management of both patients, and implications for future surgical practice.

## 2. Case Description

### 2.1. Case 1

**Figure 1:**
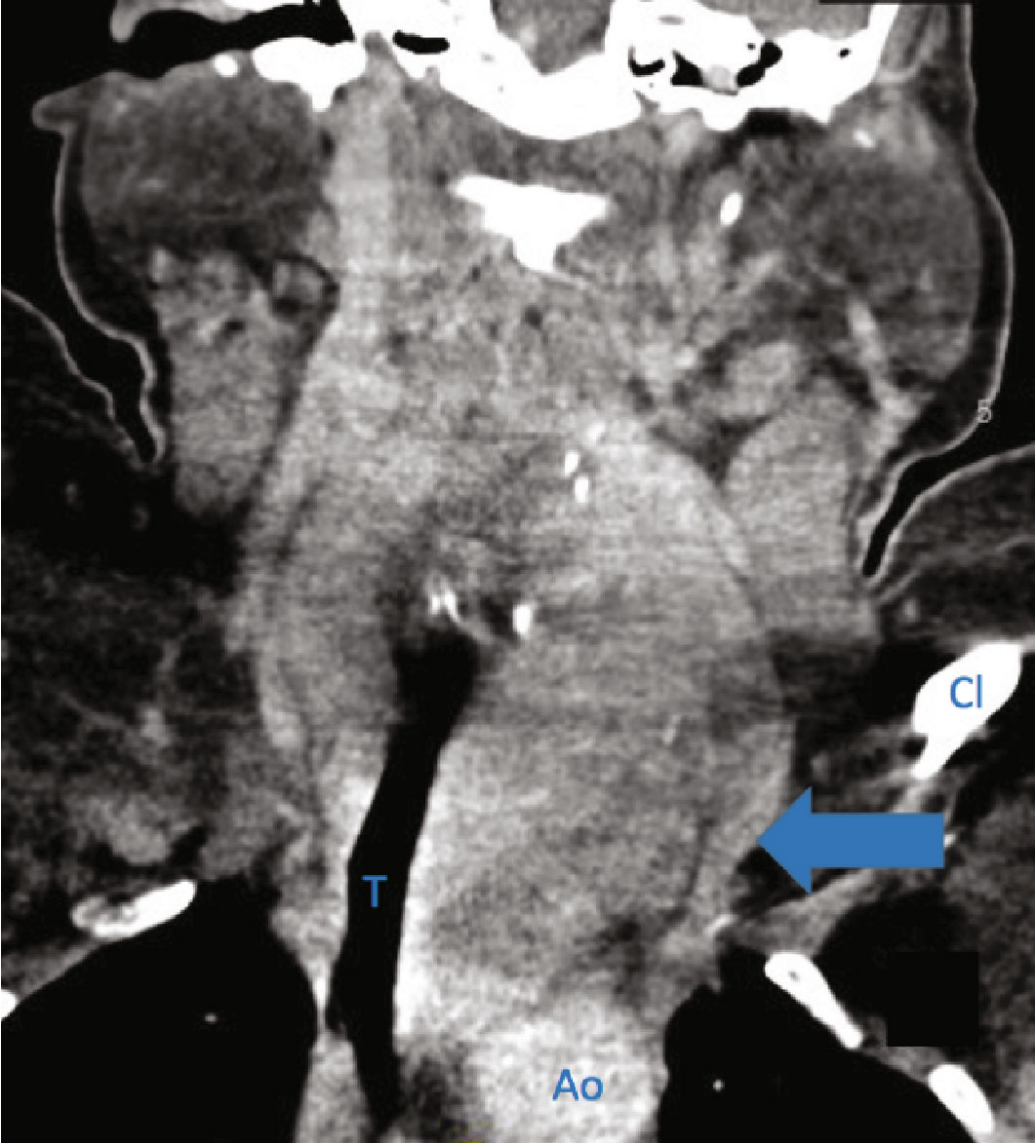
CT neck soft tissue with contrast showing bilateral thyroid goiter with substernal left thyroid extension (blue arrow), rightward tracheal deviation, and mild narrowing. Labels in picture: T: trachea; Ao: aorta; Cl: clavicle.

**Figure 2:**
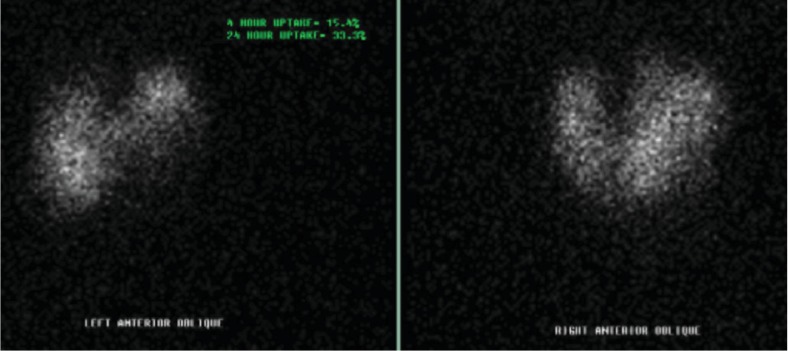
Thyroid uptake (I-123) scintigraphy showing elevated radioactive iodine uptake at 4 and 24 hours consistent with hyperthyroidism and a cold defect in the inferior pole of an enlarged left lobe for which thyroid carcinoma cannot be excluded.

A 43-year-old female was referred and evaluated in clinic with a chief complaint of a slowly enlarging neck mass. She had a past medical history of hypertension, morbid obesity, sleep apnea, and asthma. She was afebrile and denied any dyspnea, chest pain, or dysphagia. Focused physical exam noted a palpable left neck mass that moves with deglutition without clinical tracheal deviation. Upon review of her preoperative workup, her thyroid ultrasound revealed an enlarged heterogeneous left lobe of the thyroid. Computerized tomography (CT) imaging of the neck and thorax revealed a bilateral multinodular thyroid goiter with a left substernal anterior mediastinal extension and a 12.8 mm calcification on the left lower area. The adjacent trachea showed narrowing and rightward displacement due to the thyroid mass (Figure 1). A thyroid uptake (Iodine- (I-) 123) scintigraphy showed a cold defect in the inferior pole of an enlarged left lobe (Figure 2).

Ultrasound-guided fine-needle aspiration cytology (US-FNAC) of the left thyroid nodule reported a possible papillary carcinoma.

**Figure 3:**
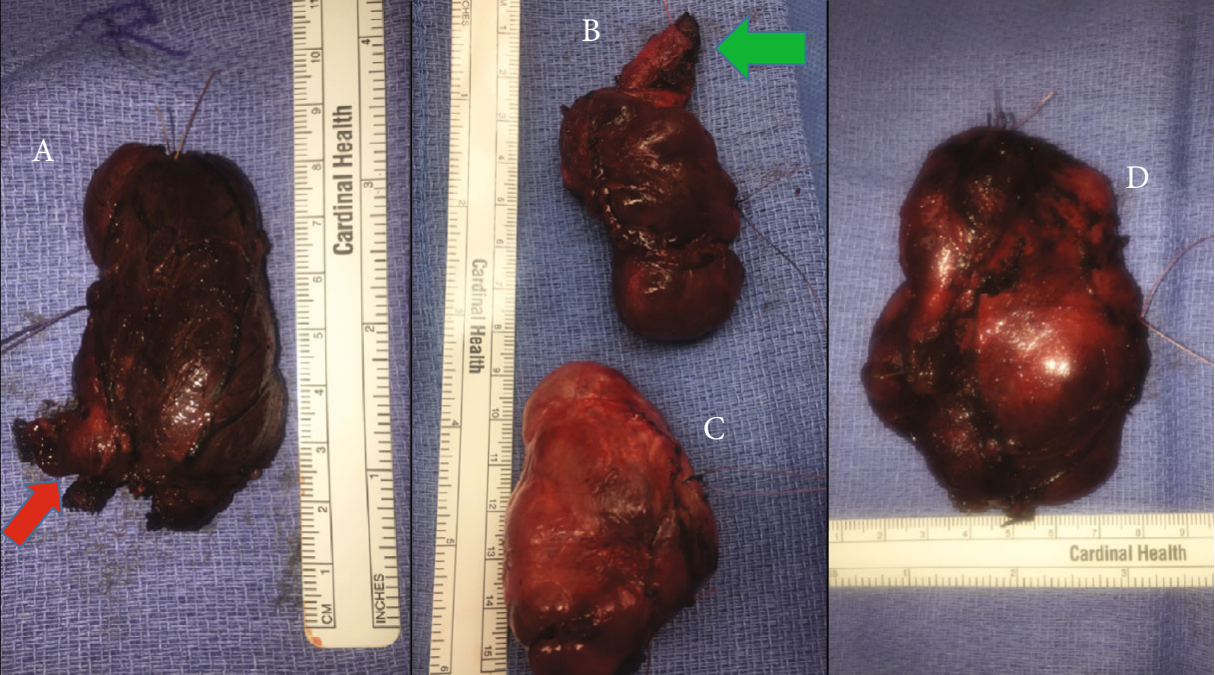
(A) The right multinodular thyroid lobe with inferior pole tail-like extension measuring 8 × 4 × 3 cm. Note the tail-like extended protrusion (red arrow). (B) Posterior ectopic thyroid measuring 8 × 5 × 2 cm. Note the teardrop shape resembling a remnant tract on the superior end (green arrow). (C) Substernal ectopic thyroid measuring 6 × 5 × 3.5 cm. (D) Left thyroid lobe measuring 8 × 5 × 2 cm.

Her operation started with a left thyroidectomy which was then converted to a total thyroidectomy with the use of intraoperative nerve monitoring (IONM) and IOUS. The left thyroid lobe was dissected with difficulty due to its larger left lobar size (10 × 7 × 5 cm), hypervascularity, and multinodularity (Figure 3). It was dissected meticulously and bluntly and was completely excised close to its capsule. The plane of dissection was clean, smooth, and left with no visible residual mass. After removal of the left thyroid, palpation revealed additional masses. A separate large left substernal mass (6 × 5 × 3.5 cm) was identified, transcervically dissected, and completely excised. Another large left mass was palpated directly posterior to the previously removed left lobe. The fat plane was opened up. This mass was also meticulously dissected and completely excised. This posteriorly located mass characteristically had a teardrop shape which appeared as a long remnant tract on the superior end. The mass measured about 8 × 5 × 2 cm and compressed laterally the carotid sheath. All of these masses were safely and completely excised (Figure 3). The recurrent laryngeal nerve (RLN) on the left was identified, well visualized, protected, and left intact with the aid of IONM. On further intraoperative evaluation including IOUS, the right thyroid lobe was identified as enlarged, hypervascular, and with palpable nodules. The pyramidal lobe was also observed as prominently enlarged. The lower pole of the thyroid was noted to have a tail-like extended protrusion distinctive of a persistent grade II thyroid rest. All enlarged lobes were dissected en bloc with the pyramidal lobe. The right multinodular thyroid lobe measured 8 × 4 × 3 cm in size (Figure 3).

A French #10 Jackson-Pratt drain was applied. She was discharged a few days later with supplemental prescriptions of calcium, vitamin D3, and levothyroxine.

### 2.2. Case 2

**Figure 4:**
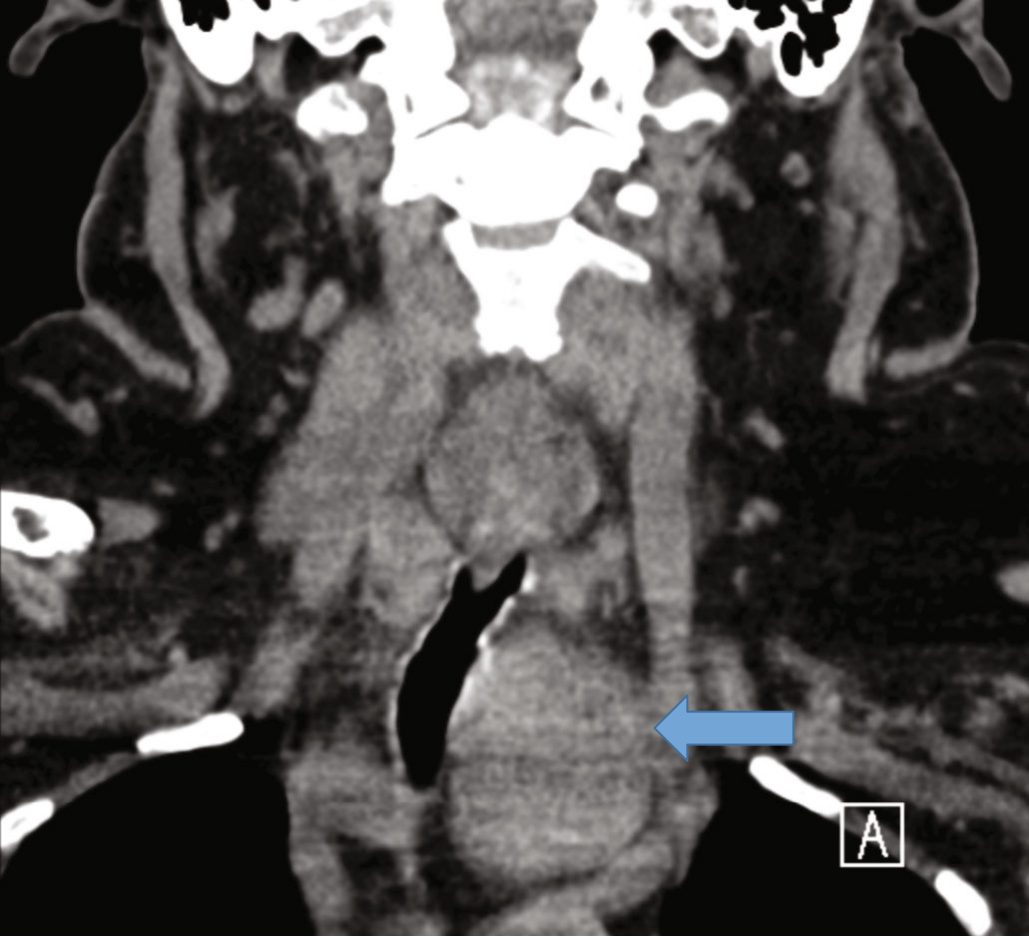
Coronal view of CT neck showing a substernal anterior mediastinal exophytic mass (blue arrow) found to be a benign thyroid mass by US-FNAC measuring 9 × 4 × 3 cm.

A 65-year-old female presented with a past medical history of hypertension, asthma, hypothyroidism, and GERD. Past surgical history included a right breast lumpectomy, cholecystectomy, 4 cesarian sections, hysterectomy, and a left thyroid lobectomy 4 years ago. She was referred after a CT scan was done which showed an incidental finding of a left substernal anterior mediastinal exophytic mass measuring 9 × 4 × 3 cm with rightward tracheal deviation (Figure 4). She was euthyroid and denied any compressive aerodigestive signs and symptoms. US-FNAC was consistent with a left substernal thyroid mass. A well-planned elective substernal thyroidectomy through median sternotomy was scheduled thereafter.

## 3. Discussion

Surgical removal of an enlarged thyroid is needed when obstructive symptoms are present or with a high suspicion for malignancy. The incidence of asymptomatic patients with thyroid nodules is increasing due availability of imaging such as ultrasound [6]. Management of an enlarged thyroid depends on American Thyroid Association recommendations based on characteristics of the nodule and the patient's age, sex, radiation exposure, and other factors [7]. Radioiodine ablation is a nonsurgical option in both benign and malignant diseases with long-term complications including hypothyroidism [8].

Ectopic thyroid has been diagnosed positively by clinicians through preoperative imaging in our review of the literature [9]. Surgeons must be well adept in the knowledge of thyroid and parathyroid embryogenesis, with its diverse aberrance of embryologic, histologic, and anatomic development. All of this knowledge coupled with the surgeon's experience and intuition is of utmost importance intraoperatively to prevent a retained or missed thyroid, especially if positive for malignancy.

Our first surgical case was intraoperatively interesting. Two left ectopic thyroid masses were found. A left substernal mass and another mass posterior to the left thyroid were developed. The right thyroid with its tail-like extension or protuberance was also considered aberrant. Only on further inspection and palpation along with IOUS were further ectopic thyroid masses and aberrant thyrothymic rests identified. Thus, in all partial or complete thyroidectomy cases, surgeons should appreciate the normal anatomy of the thyroid. Moreover, the knowledge of embryogenesis is of utmost importance in recognizing ectopic thyroid masses. Knowledge of embryogenesis and anatomy is required to excise ectopic glands during the same operation, preventing a retained or missed thyroid gland.

Our second case describes a patient who had a left thyroid lobectomy 4 years ago. The primary care physician ordered a neck and chest CT recently for a suspected goiter. Incidentally, a left substernal anterior mediastinal mass was found compressing and significantly deviating the trachea to the right. US-FNAC showed a thyroid mass. This was suggestive of a missed and therefore a retained ectopic thyroid (a persistent median thyroid rest anlage or an unfused aberrant lateral anlage) during the initial left thyroid lobectomy operation.

A good surgical dissection and exposure aids in a safer operation. It starts from skin incision along the Langer lines. After the avascular subplatysmal plane is dissected, transection of the midline investing layer of superficial cervical fascia is next done to expose the visceral cervical space of the neck. Lateral retraction of strap muscles is needed for wide exposure of thyroid vessels especially on the superior area to identify and preserve the superior laryngeal nerve. Identification of the Tubercle of Zuckerkandl (TZ) is mandatory with its close anatomic relationship and variability with the RLN and parathyroid gland (PtG). This will prevent RLN injury and preserve the PtG without devascularization. The TZ has four grades: #0, unrecognizable; #1, thickening of the lateral edge of the thyroid lobe; #2, less than 1 cm; and #3, greater than 1 cm [10]. Prior to closure, final post procedure reevaluation with palpation and/or use of IOUS is needed not to miss any other mass. All of these dissection steps allow good visualization of the thyroid itself and/or any ectopic aberrant thyroid.

In normal embryogenesis, thyroid anlagen migrate from the floor of the primitive foregut to its location on the midline of the neck in front of the trachea [11]. If the developing thyroid does not follow this migration, the gland will develop ectopically [1]. Over 50% of thyroid dysgenesis cases have a correlation with an ectopic thyroid. Several genetic abnormalities were associated with impaired thyroid development including FoxE1, TTF-1, TTF-2, Pax-8, and HoxA3 [12, 13]. The thyroglossal duct is attached to the foramen cecum and thyroid gland during migration and normally involutes. Although debated among embryologists, two anlagen (median and lateral) are needed in the development of the thyroid. Normally, the median and lateral anlagen fuse during embryological development. According to some embryologists, the lateral thyroid anlage is derived from the ultimobranchial body, a descending diverticulum of the fourth pharyngeal pouch (as it embryologically descends along with the inferior parathyroid) [13]. The presence of a lateral thyroid anlage can explain the nonmidline ectopic thyroid tissue in the neck, since an arrest of migration of the lateral thyroid anlage would result in the failure of fusion with the medial anlage [11]. Our posterior ectopic thyroid and substernal ectopic thyroid could represent a persistent abnormality of the lateral thyroid anlage. Embryologic abnormalities and developmental variations of median thyroid anlagen, lateral thyroid anlagen, both anlagen, and neither anlage have been detailed extensively in previous literature [12].

**Figure 5:**
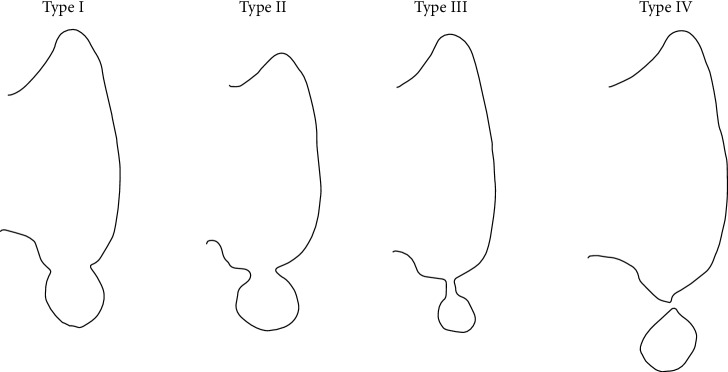
Thyroid rests types I-IV. Type I: a protrusion of thyroid tissue from the inferior of the thyroid gland and distinct from the lower border of the thyroid lobe. Type II: thyroid tissue within the thyrothymic tract and attached to the thyroid by a narrow pedicle of thyroid tissue. Type III: thyroid tissue similar to type II but attached to the thyroid gland by a fibrovascular core. Type IV: has no thyroid gland connection [14].

Thyroid rests can also form, which are isolated normal tissue below the lower pole of thyroid in the line of the thyrothymic tract or in the upper anterior mediastinum. They are remnants from the thyroid descent of the median anlage during normal embryogenesis. Our dissected right thyroid lobe has a type II thyroid rest. A classification of thyroid rests from types I-IV was proposed in relation to the thyroid (Figure 5) [14].

After removing an ectopic thyroid, pathology is needed to exclude the possibility of a well-differentiated metastasis that replaced a lymph node [15]. In our case, all tissue samples were negative for any malignancy. There are few reported cases of dual or triple ectopic thyroids [1, 16–24]. Our case was not detected on preoperative workup including CT scan, ultrasound, and thyroid scintigraphy. Excellent surgical knowledge of the anatomy and embryology of the thyroid is necessary not to cause surgical complications including bleeding, RLN injury, relative or absolute hypoparathyroidism, and a missed ectopic thyroid [25]. The first case depicts excellent neck dissection and exposure of an enlarged hypervascular thyroid, with safe preservation of RLN (with use of IONM) and PtG. After a routine thyroidectomy prior to closure, surgeons should intraoperatively palpate the operative site including the retrosternal anterior mediastinal area through the neck incision. For the same reason, surgeons should use IOUS. In the second case, an incidental CT finding of retrosternal mass after a past surgical history of left thyroid lobectomy was found to be a thyroid mass.

In summary, an experienced and intuitive neck surgeon should know the normal thyroid anatomy and its embryonic development. A surgeon should identify any congenital aberrant anomalies in the perioperative period and prevent future complications. Surgeons must keep in mind the possibility of missing an ectopic thyroid that could be malignant.
